# Imaging of pulmonary perfusion using subtraction CT angiography is feasible in clinical practice

**DOI:** 10.1007/s00330-018-5740-4

**Published:** 2018-09-25

**Authors:** Dagmar Grob, Luuk J. Oostveen, Mathias Prokop, Cornelia M. Schaefer-Prokop, Ioannis Sechopoulos, Monique Brink

**Affiliations:** 1 Department of Radiology and Nuclear Medicine, Radboud University Medical Center, Geert Grooteplein 10, 6525 GA Nijmegen, The Netherlands; 2 Department of Radiology and Nuclear Medicine, Meander Medical Centre, Maatweg 3, 3813 TZ Amersfoort, The Netherlands

**Keywords:** Subtraction technique, Computed tomography scanner, Contrast media, Perfusion imaging, Pulmonary embolism

## Abstract

**Abstract:**

Subtraction computed tomography (SCT) is a technique that uses software-based motion correction between an unenhanced and an enhanced CT scan for obtaining the iodine distribution in the pulmonary parenchyma. This technique has been implemented in clinical practice for the evaluation of lung perfusion in CT pulmonary angiography (CTPA) in patients with suspicion of acute and chronic pulmonary embolism, with acceptable radiation dose. This paper discusses the technical principles, clinical interpretation, benefits and limitations of arterial subtraction CTPA.

**Key Points:**

*• SCT uses motion correction and image subtraction between an unenhanced and an enhanced CT scan to obtain iodine distribution in the pulmonary parenchyma.*

*• SCT could have an added value in detection of pulmonary embolism.*

*• SCT requires only software implementation, making it potentially more widely available for patient care than dual-energy CT.*

## Introduction

Imaging techniques have been developed over the years to study pulmonary perfusion, not only as a tool to investigate the sequelae of vascular obstruction, such as acute and chronic pulmonary embolism (PE), but lately also as a potential tool to characterise inflammation and the malignancy potential of lung lesions [1–3].

As early as 1964, nuclear medicine has been able to assess pulmonary perfusion using isotopes that accumulate in the capillary bed [4]. Although all modern nuclear medicine methods can accurately quantify true perfusion in the pulmonary parenchyma, they also have important drawbacks, such as a low spatial resolution, issues with isotope availability, production and handling, and high cost [5].

Magnetic resonance imaging (MRI) and computed tomography (CT) are more widely available, but are not capable of imaging true substance exchange. Total intravascular movement can be measured with MRI using arterial spin labelling techniques. In addition, volume and speed of vascular contrast distribution are measured with dynamic magnetic resonance angiography [6]. MRI does not use ionising radiation, and has a better spatial resolution than nuclear imaging techniques, but its images are frequently technically inadequate because of a low signal-to-noise ratio, susceptibility and motion artefacts due to air and respiratory movement, and the low amount of tissue in the lungs [2, 7]. Therefore, MRI is not used as a primary imaging tool of the pulmonary vasculature in most hospitals. CT is far more widely accepted as the modality of choice for evaluation of the lungs because of its higher spatial and temporal resolution [8, 9] and its new ability to display iodine distribution, reflecting pulmonary perfusion.

A CT technique commonly used in clinical practice for assessing pulmonary perfusion is dual-energy CT (DECT). It uses material decomposition of iodine from other materials to visualise the regional pulmonary distribution of intravenous contrast in the pulmonary vessels, including the capillaries [10]. This is accomplished by almost simultaneously irradiating the patient with two x-ray beams of different energy, or by using spectral detectors and then processing the data to generate iodine maps, at a radiation dose similar to or moderately higher than CT pulmonary angiography (CTPA) [11]. This technique can show PE-associated perfusion defects (PD) [12, 13] in concordance with ventilation-perfusion single-photon emission computed tomography (V/Q SPECT) findings [14], with increased the sensitivity in detection of PE at CTPA [12, 15]. However, DECT requires dedicated dual-energy hardware.

An alternative, yet far less widely implemented CT technique to evaluate pulmonary perfusion alongside CTPA is subtraction CT (SCT), made possible by a post-processing technique that does not require special hardware. This technical note will introduce the concept of SCT, along with its clinical interpretation, benefits, limitations, and future perspectives.

## Technical principle

SCT involves the subtraction of an unenhanced, pre-injection CT image from an enhanced, post-injection CT image to obtain information on iodine distribution. Since every CT scanner is capable of making an unenhanced and enhanced CT scan, the technique would be more widely applicable than DECT. SCT was introduced by Screaton in 2003 and Wildberger in 2005 [16, 17]. Both studies showed clear visualisation of perfusion defects due to vascular obstruction in anesthetised animals, which guaranteed almost no motion between the two scans.

**Fig. 1:**
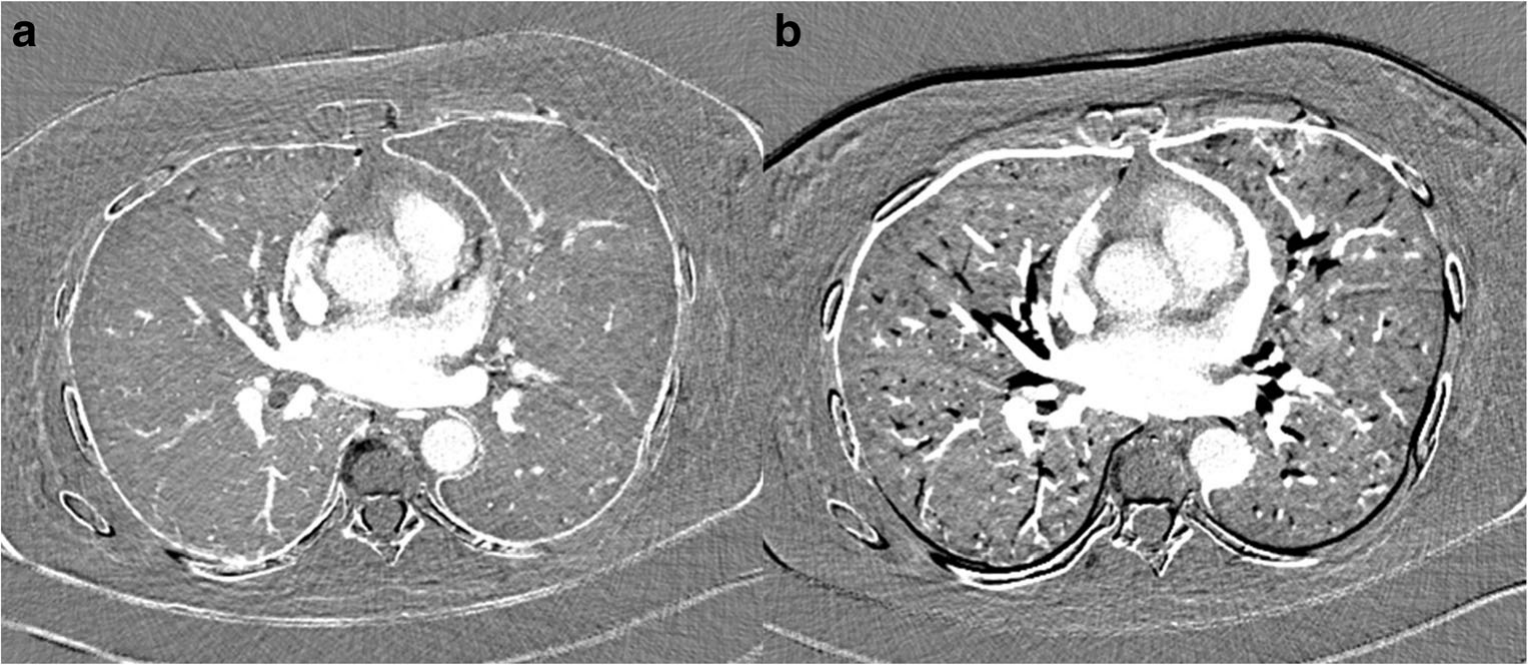
1-mm axial CTPA reconstructions after subtraction of an unenhanced CT from a CTPA with a diaphragm difference of 11 mm between the scans. **a** With motion correction, and (**b**) without motion correction

In phantom experiments with different iodine densities, SCT showed a higher contrast-to-noise ratio between soft tissue and iodine compared to DECT. In DECT, the signal difference between the low- and high-energy images is roughly linearly associated with the local iodine concentration. This implies that the signal of the high-energy image is actually subtracted from the low-energy image, reducing the signal in DECT compared to SCT, where the iodine signal is fully exploited [18, 19]. However, subject motion between the two acquisitions in SCT must be absent or compensated for to result in only the iodine signal. This means that motion correction is the biggest challenge in SCT. With increasing performance and speed of registration algorithms, achieving adequate motion correction is now feasible (Fig. 1). In our practice, we use motion correction software for SCT (^SURE^Subtraction Lung, Canon Medical Systems) that employs an iterative, non-rigid registration framework to register the unenhanced to the enhanced CT images [20]. This means that both linear and non-linear voxel displacements can be registered. Experiments with dynamic digital phantoms demonstrated that this software can correct motion adequately. Specifically, in simulated scans involving a caudo-cranial diaphragm position difference of 20 mm between the unenhanced and enhanced scans, the 75^th^ percentile of the lung voxel-to-voxel residual error distance was 1.6 mm [21]. This seems sufficient for evaluating clinically relevant perfusion defects caused by segmental and potentially first-order subsegmental vascular obstructions, as perfusion defects caused by subsegmental embolism are in the centimetre range [16], and the median diaphragm difference between unenhanced and enhanced CT scans in our clinical practice is 5.7 mm in CTPA scans.

## Subtraction in clinical practice

### Image acquisition

SCT has been added to CTPA for all adult patients with suspicion of PE at our institution for the last 3 years. CT examinations are performed on a 320-multislice detector row CT system (Aquilion ONE GENESIS and VISION, Canon Medical Systems) according to the protocol in Table 1. In order to avoid DECT-like artefacts, it is crucial that the tube voltages for the enhanced and unenhanced scans are equal [22]. Both the unenhanced and the enhanced scan are acquired during a shallow breath-hold. To obtain an optimal CTPA enhancement, 60 ml of intravenous contrast is administered with an iodine concentration of at least 300 mg/ml and bolus triggering in the pulmonary artery, in order to guarantee sufficient contrast circulation in the pulmonary parenchyma.

**Table 1:** Example of an SCTPA protocol

Acquisition/injection	Specific settings
Pre-contrast CT	Exposure parameters: 100 kV, automatic exposure control (SD 35)
	Scan parameters: cranio-caudal scan with 80 × 0.5 mm collimation, pitch 0.8 0.275 s rotation time, shallow breath-hold
	Reconstruction: 1 mm sections with 0.8 mm increment,
	3rd-generation iterative reconstruction (AIDR-3D enhanced)
Contrast injection	60 ml iodinated contrast (300 mg/ml) + 40 ml saline chaser @ 5 ml/s via a 20G needle in the left arm
Bolus triggering	ROI placement on pulmonary trunk, level circa 1 cm below carina, absolute threshold: 220 HU. After reaching the threshold there is 5 s scan delay. The related software for automatic bolus triggering is ^SURE^Start
Post-contrast CT	Exposure parameters: 100 kV, automatic exposure control (SD 22.5)
	Scan parameters: cranio-caudal scan with 80 × 0.5 mm collimation, pitch 0.8 0.275 s rotation time, shallow breath-hold
	Reconstruction: 1 mm sections with 0.8 mm increment, 3rd-generation iterative reconstruction (AIDR-3D enhanced)

### Radiation dose

In our hospital, the median dose-length product (DLP) of this protocol in patients scanned between August 2016 and January 2017 (n = 354 patients) was 191 mGy∙cm (mean DLP: 266 mGy∙cm) , with a median DLP of 59 mGy∙cm (mean: 74 mGy∙cm) for the unenhanced scan and of 122 mGy∙cm (mean: 183 mGy∙cm) for the enhanced scans. The effective dose was 2.8 mSv, which is calculated from the total median DLP multiplied by 0.0146 mSv/(mGy∙cm) [23]. This is lower than the average dose of a CTPA scan (3–5 mSv) [8]. In 55 patients who underwent both DECT and SCT in a prospective study, the whole subtraction protocol was executed with a lower radiation dose than DECT, while subjectively evaluated image quality was better [24].

### Reconstruction

**Fig. 2:**
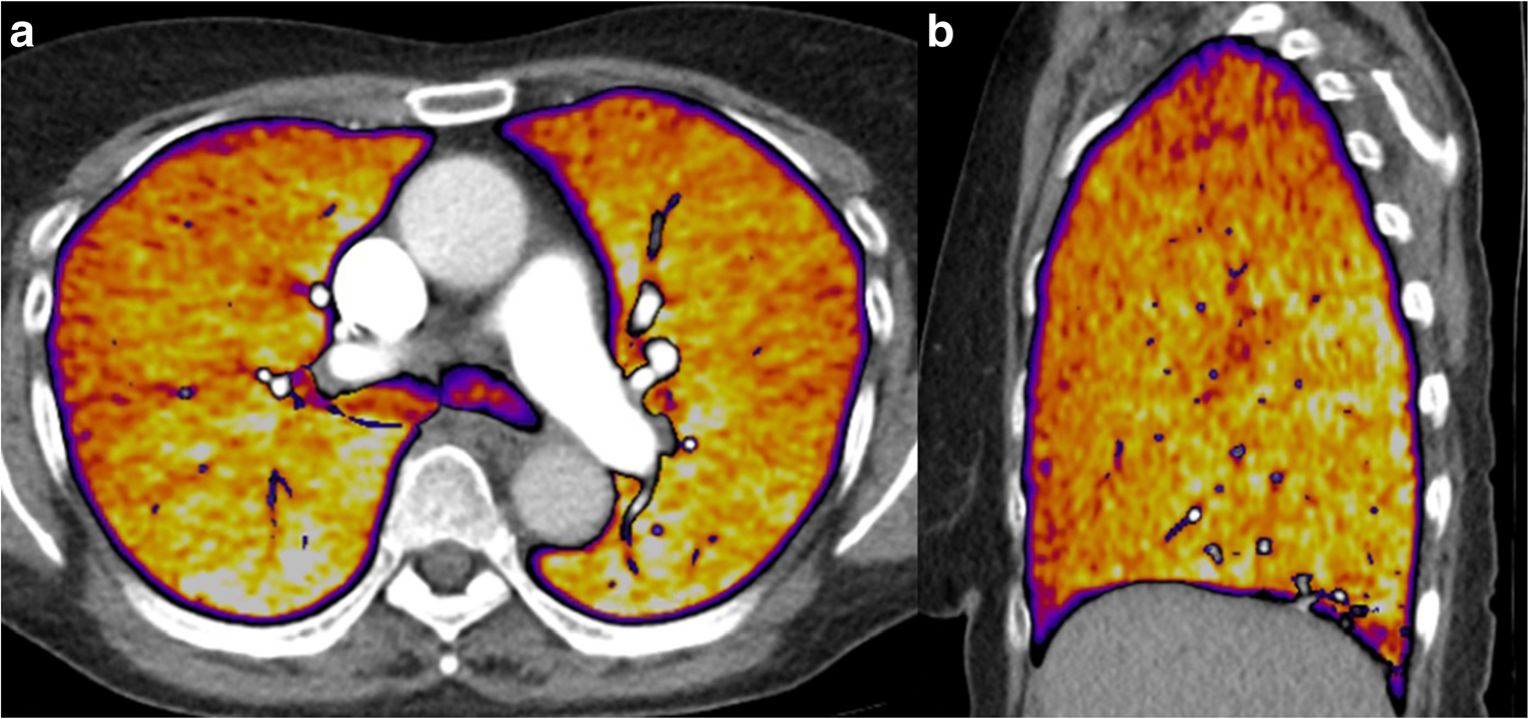
5-mm (**a**) axial and (**b**) sagittal reconstructions of a subtraction iodine map on top of CTPA of normally-perfused lungs in a supine position. Both reconstructions show a normal gravity-dependent gradient, in the ventro-dorsal and the cranio-caudal direction

The subtraction software automatically selects the unenhanced and enhanced scans, applies a mask to extract only the lung areas and registers and deforms the lungs in the unenhanced scan to their shape and position in the enhanced scan. After subtraction, it automatically generates 1-mm greyscale iodine maps of the lungs with exclusion of the large vessels and 5-mm heat scale colour maps as an overlay on top of the CTPA images. These maps reflect true Hounsfield unit density differences in the pulmonary parenchyma between the two scans, with a pre-set WW/WL of 100/50 (Fig. 2). It is possible to reconstruct afterwards images at their own preference thickness, for example, even thicker slices, like 10 mm.

### Interpretation

**Fig. 3:**
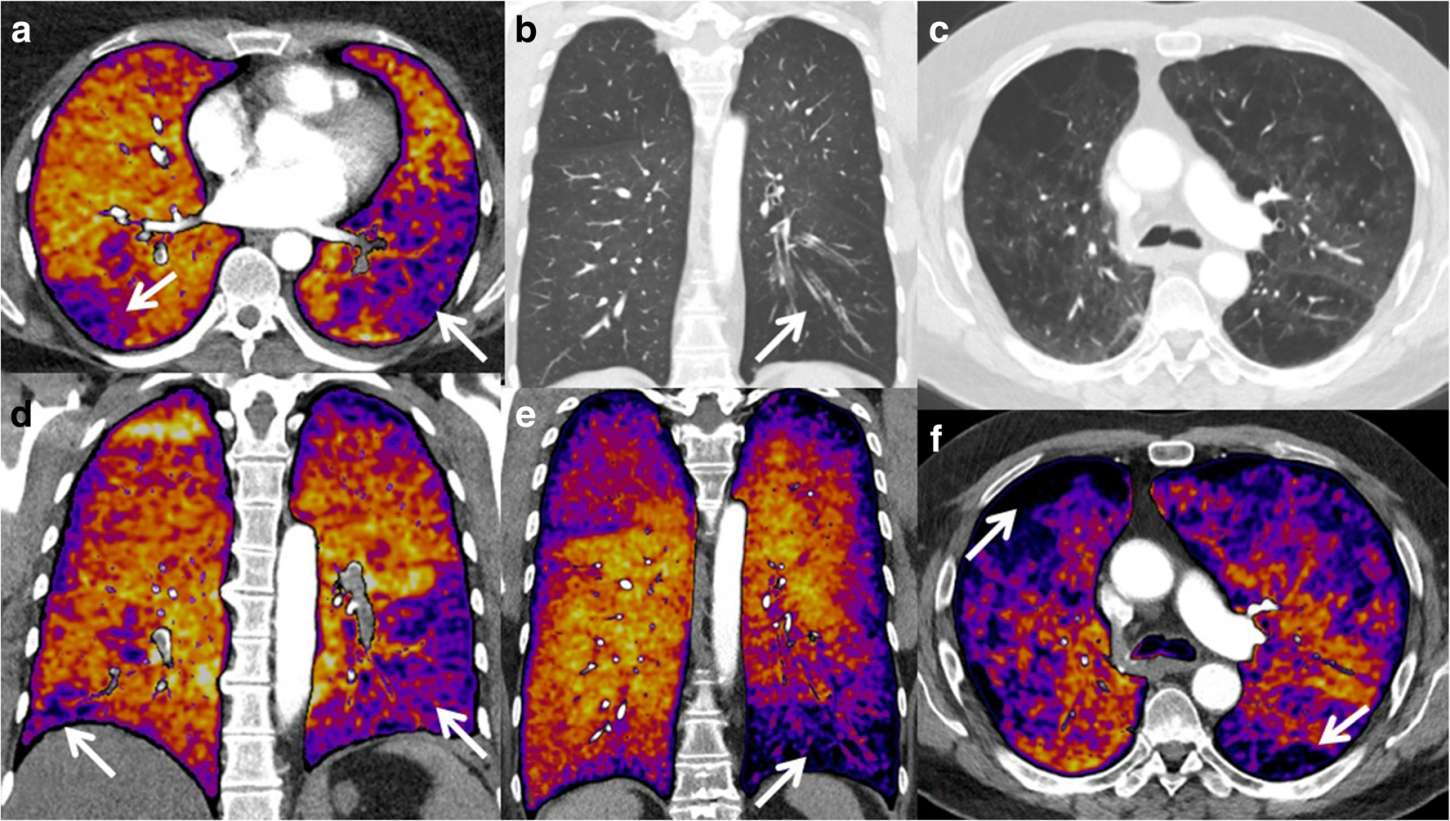
**a**, **d** 3-mm slices of a patient with bilateral lobar pulmonary embolism and corresponding wedge-shaped perfusion defects in both lungs (arrows). **b** 3-mm slices of CTPA in a lung window, and, (**e**) a coronal view with iodine map of a patient with left lower lobe bronchopathy with mucous plugging (arrow) and corresponding perfusion defects (arrows). **c**, **f** 3-mm reconstruction of CTPA and subtraction maps of a patient with predominant centrilobular emphysema; the destroyed pulmonary parenchyma does not show iodine uptake (arrows)

Subtraction perfusion maps can only be used complementary to CTPA. Initial evaluation of the maps in three directions facilitates appreciation of the normal ventro-dorsal gradient of pulmonary blood volume in the supine patient [25], and easier recognition of artefacts. In addition, this initial review allows for a more accurate assessment of the shape of potential perfusion inhomogeneities [26], which allows for differentiation of the typical triangular shape of perfusion inhomogeneities due to vascular pathology or bronchopathy from pathologies such as emphysema (Fig. 3). To distinguish these entities, scrutiny of the CTPA is necessary to rule out PE, while evaluation of the bronchial system and lung parenchyma is necessary to rule out bronchial abnormalities or emphysema. Because perfusion inhomogeneities due to vascular or bronchial disease affect structures larger than the secondary lobules, which are within the centimetre range, reconstruction of thick multiplanar reconstructions of 5–10 mm or are sufficient for initial interpretation. One-millimetre-thick slices can be used to assess smaller structures such as subsegmental PE, but at the expense of the increased noise and a higher susceptibility to the effects of inaccurate motion correction.

### Artefacts

**Fig. 4:**
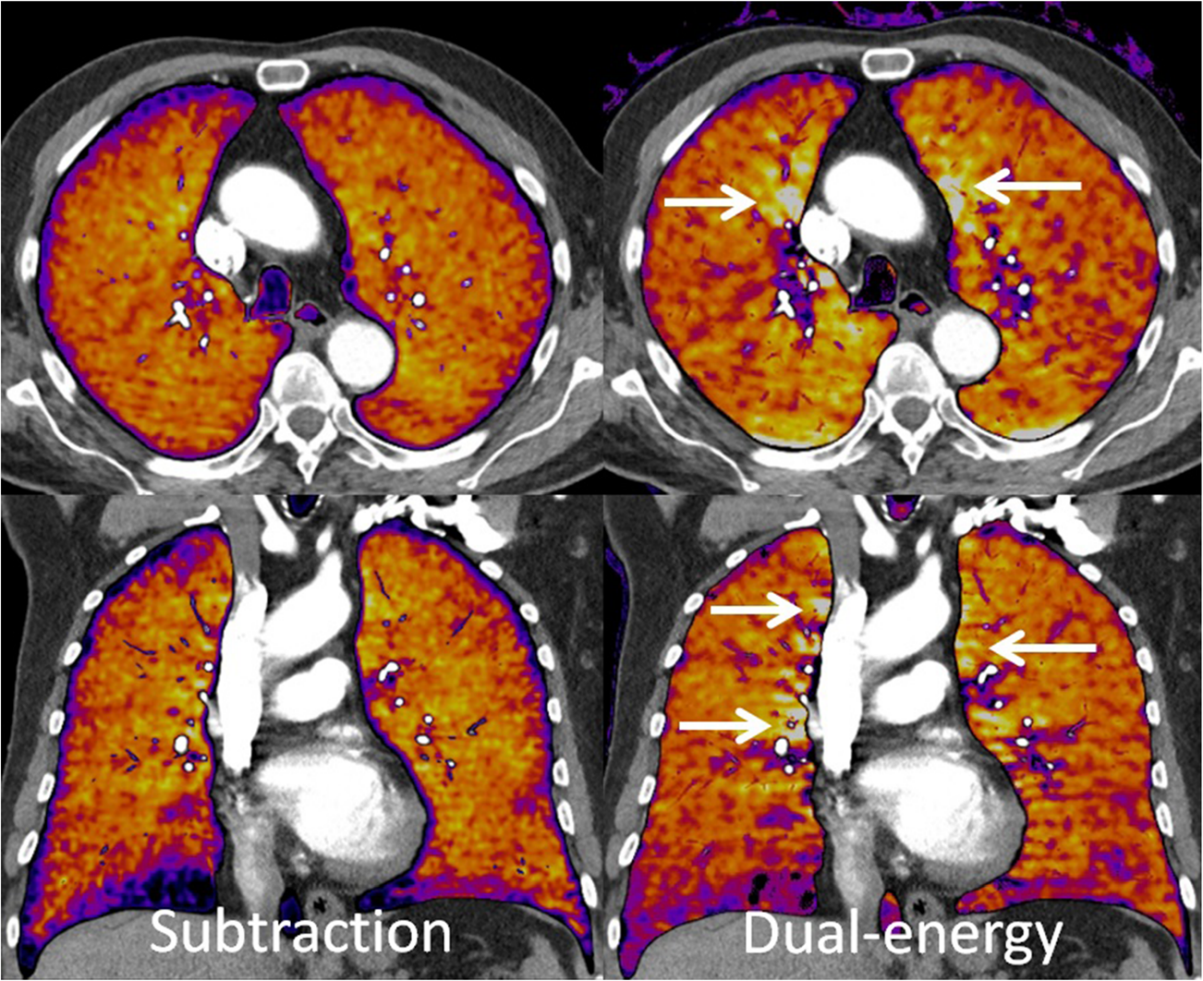
3-mm axial and coronal slices of subtraction iodine maps and dual-energy iodine maps, both obtained from a dual-source scanner. The arrows show typical beam-hardening artifacts that are more severe in dual-energy than in subtraction iodine maps

Beam-hardening artefacts may occur close to the high-density contrast column next to the superior caval vein in cases where a high-concentration of contrast material is still present in the injection veins [13]. These artefacts are less severe than those encountered in dual-source-based dual-energy techniques (Fig. 4) [24]. The probable reason is that the two tubes image the same structures with a time delay that is roughly a quarter of the rotation time, which might be enough for substantial variations in local contrast concentration in the inflow veins [13].

The images need to be evaluated in axial reconstruction but also in coronal or sagittal reconstructions to recognise artefacts. Motion artefacts can occur in SCT if lung volume and position, diaphragm position or cardiac pulsation significantly differ between the scans, even after registration. The type of artefact predominantly occurs in the basal lung fields, slightly above the diaphragm, and in the paracardiac region [21]. Presence of these artefacts can be assessed by checking differences between the enhanced and unenhanced scans on coronal reconstructions and producing 10-mm MinIP from the subtraction iodine maps. In case of severe motion correction flaws, black lines parallel to the vasculature will appear. The thicker the line, the worse the artefacts are.

Suboptimal contrast enhancement of the lung shows low-density areas in the iodine maps that can hamper the diagnostic evaluation. However, adapting the WW/WL of the greyscale and colour heat map can help.

## Discussion

SCT is a simple, inexpensive and fast method for imaging and relative quantification of pulmonary perfusion at an acceptable radiation dose. The technique has been implemented in clinical practice in patients with suspicion of pulmonary embolism for several years, while having potential for other clinical applications that evaluate enhancement of the pulmonary parenchyma.

Iodine maps created with SCT are similar to those created with DECT. Artefact behavior is different, however; DECT images suffer from motion artefacts if some of the anatomy in the field of view moves during or between the high- or low-energy scan. The latter effect is non-existent for energy-discriminating detectors, and negligible for a rapid kV-switching system, but could play a role in dual-source systems or systems with dual rotation [18]. Motion artefacts in SCT occur if the patient moves during one of the two scans or if the inspirational difference between scans is major and is not adequately addressed. Streak artefacts due to contrast inflow are less severe with SCT compared to dual-source DECT [24].

SCT- and DECT-derived iodine maps reflect the iodine enhancement at the moment of image acquisition. However, absolute quantification of pulmonary perfusion parameters is impossible to achieve from a single acquisition, be it SCT or DECT, and requires sequential scanning (CT perfusion). This is because the variability in contrast enhancement among patients is high when a single acquisition is evaluated. The pulmonary contrast enhancement not only depends on the presence of pathology and contrast administration parameters, but also on parameters such as respiratory cycle dynamics, cardiac output, and bronchial physiology [27, 28]. In addition, the enhancement pattern in the pulmonary parenchyma may change with time after contrast arrival, especially if there is bronchial arterial collateral supply to areas of the lung that are hypo-perfused by the pulmonary artery [29]. A second scan in the systemic arterial phase can help distinguish these two perfusion components. If sequential scanning were performed at a reasonable dose, absolute quantification might be possible. This might result in the ability to obtain functional (perfusion) information of structures in the pulmonary parenchyma. Other possibilities could be to characterise small structures or lesions, e.g. nodules, with the potential benefit of distinguishing between benign and malignant nodules [30].

Improvements in SCT could be achieved by reducing motion artefacts due to cardiac motion, pulsation and breathing in the pre- and post-contrast images using electrocardiogram (ECG) synchronisation or advanced motion-correction techniques. Improved registration of small vessels would provide not only subtraction imaging of the lung parenchyma, but of the vasculature itself [31]. This can be helpful for direct clot detection (lack of iodine enhancement causes a defect in the vessel on iodine maps). Improved beam hardening would help eliminate streak artefacts due to high contrast concentration in inflow veins [32].

In conclusion, software-based motion correction combined with temporal subtraction enables imaging of contrast enhancement in the lungs similar to DECT. The technique is widely applicable at a radiation dose equivalent to DECT. Similar to DECT, SCT depicts perfusion abnormalities in patients with vascular, bronchial or other pathology. Future advances in pulmonary SCT include dynamic acquisition and reduction of motion artefacts in small structures.

## Abbreviations

CT: Computed tomography
CTPA: Computed tomography pulmonary angiography
DECT: Dual-energy computed tomography
DLP: Dose length product
MRI: Magnetic resonance imaging
PE: Pulmonary embolism
SCT: Subtraction computed tomography

## References

[1] Chae EJ, Song JW, Krauss B (2010). Dual-energy computed tomography characterization of solitary pulmonary nodules. J Thorac Imaging.

[2] Hopkins SR, Wielpütz MO, Kauczor HU (2012) Imaging lung perfusion. J Appl Physiol (1985) 113:328–339 10.1152/japplphysiol.00320.2012 PMC3404706 22604884

[3] Mistry NN, Pollaro J, Song J, De Lin M, Johnson GA (2008) Pulmonary perfusion imaging in the rodent lung using Dynamic Contrast Enhanced MRI. Magn Reson Med 59:289–297 10.1002/mrm.21353 PMC2738602 18228577

[4] Elgazzar AH (2015). The Pathophysiologic Basis of Nuclear Medicine, 3 edn.

[5] Dubsky S, Fouras A (2015). Imaging regional lung function: a critical tool for developing inhaled antimicrobial therapies. Adv Drug Deliv Rev.

[6] Haller S, Zaharchuk G, Thomas DL, Lovblad KO, Barkhof F, Golay X (2016). Arterial Spin Labeling Perfusion of the Brain: Emerging Clinical Applications. Radiology.

[7] Ley-Zaporozhan J, Ley S, Eberhardt R (2007). Assessment of the relationship between lung parenchymal destruction and impaired pulmonary perfusion on a lobar level in patients with emphysema. Eur J Radiol.

[8] Remy-Jardin M, Pistolesi M, Goodman LR (2007). Management of suspected acute pulmonary embolism in the era of CT angiography: a statement from the Fleischner Society. Radiology.

[9] Patel S, Kazerooni EA, Cascade PN (2003). Pulmonary embolism: optimization of small pulmonary artery visualization at multi-detector row CT. Radiology.

[10] Johnson TR, Krauss B, Sedlmair M (2007). Material differentiation by dual energy CT: initial experience. Eur Radiol.

[11] Thieme SF, Johnson TR, Lee C (2009). Dual-energy CT for the assessment of contrast material distribution in the pulmonary parenchyma. AJR Am J Roentgenol.

[12] Pontana F, Faivre JB, Remy-Jardin M (2008). Lung perfusion with dual-energy multidetector-row CT (MDCT): feasibility for the evaluation of acute pulmonary embolism in 117 consecutive patients. Acad Radiol.

[13] Kang MJ, Park CM, Lee CH, Goo JM, Lee HJ (2010). Dual-energy CT: clinical applications in various pulmonary diseases. Radiographics.

[14] Meysman M, Everaert H, Buls N, Nieboer K, de Mey J (2015). Comparison of ventilation-perfusion single-photon emission computed tomography (V/Q SPECT) versus dual-energy CT perfusion and angiography (DECT) after 6 months of pulmonary embolism (PE) treatment. Eur J Radiol.

[15] Okada M, Kunihiro Y, Nakashima Y (2015). Added value of lung perfused blood volume images using dual-energy CT for assessment of acute pulmonary embolism. Eur J Radiol.

[16] Screaton NJ, Coxson HO, Kalloger SE (2003). Detection of lung perfusion abnormalities using computed tomography in a porcine model of pulmonary embolism. J Thorac Imaging.

[17] Wildberger JE, Klotz E, Ditt H, Spuntrup E, Mahnken AH, Günther RW (2005). Multislice computed tomography perfusion imaging for visualization of acute pulmonary embolism: animal experience. Eur Radiol.

[18] Faby S, Kuchenbecker S, Sawall S (2015). Performance of today's dual energy CT and future multi energy CT in virtual non-contrast imaging and in iodine quantification: A simulation study. Med Phys.

[19] Baerends E, Oostveen LJ, Smit CT et al (2018) Comparing dual energy CT and subtraction CT on a phantom: which one provides the best contrast in iodine maps for sub-centimetre details? Eur Radiol. 10.1007/s00330-018-5496-x 10.1007/s00330-018-5496-x PMC6223839 29808430

[20] Goatman K, Plakas C, Schuijf J, Beveridge E, Prokop M (2014) Computed tomography lung iodine contrast mapping by image registration and subtraction. Proc of SPIE 9034, Medical Imaging 2014: Image Processing, 10.1117/12.2043551

[21] Grob D, Oostveen LJ, Rühaak J et al (2018) *under review -* Accuracy of registration algorithms in subtraction CT of the lungs: a digital phantom study 10.1002/mp.13496 PMC6849605 30888690

[22] Winklhofer S, Lambert JW, Sun Y, Wang ZJ, Sun DS, Yeh BM (2016). Pelvic Beam-Hardening Artifacts in Dual-Energy CT Image Reconstructions: Occurrence and Impact on Image Quality. AJR Am J Roentgenol.

[23] Deak PD, Smal Y, Kalender WA (2010). Multisection CT protocols: sex- and age-specific conversion factors used to determine effective dose from dose-length product. Radiology.

[24] Grob D, Smit EJ, Oostveen LJ et al (2017) Intra-individual comparison of direct subtraction vs. dual-energy for imaging of pulmonary perfusion- a feasibility study. European Congres of Radiology (ECR) 2017, Vienna

[25] Almquist HM, Palmer J, Jonson B, Wollmer P (1997). Pulmonary perfusion and density gradients in healthy volunteers. J Nucl Med.

[26] Roach PJ, Bailey DL, Schembri GP, Thomas PA (2010). Transition from Planar to SPECT V/Q Scintigraphy: Rationale, Practicalities, and Challenges. Semin Nucl Med.

[27] Takx RAP, Henzler T, Schoepf UJ (2017). Predictive value of perfusion defects on dual energy CTA in the absence of thromboembolic clots. J Cardiovasc Comput Tomogr.

[28] Meinel FG, Graef A, Sommer WH, Thierfelder KM, Reiser MF, Johnson TR (2013). Influence of vascular enhancement, age and gender on pulmonary perfused blood volume quantified by dual-energy-CTPA. Eur J Radiol.

[29] Koike H, Sueyoshi E, Sakamoto I, Uetani M (2017). Clinical Significance of Late Phase of Lung Perfusion Blood Volume (Lung Perfusion Blood Volume) Quantified by Dual-Energy Computed Tomography in Patients With Pulmonary Thromboembolism. J Thorac Imaging.

[30] Ohno Y, Nishio M, Koyama H (2015). Solitary pulmonary nodules: Comparison of dynamic first-pass contrast-enhanced perfusion area-detector CT, dynamic first-pass contrast-enhanced MR imaging, and FDG PET/CT. Radiology.

[31] Murphy K, van Ginneken B, Reinhardt JM (2011). Evaluation of registration methods on thoracic CT: the EMPIRE10 challenge. IEEE Trans Med Imaging.

[32] Barrett JF, Keat N (2004). Artifacts in CT: recognition and avoidance. Radiographics.

